# A Correlation among the COVID-19 Spread, Particulate Matters, and Angiotensin-Converting Enzyme 2: A Review

**DOI:** 10.1155/2021/5524098

**Published:** 2021-05-11

**Authors:** Zafran Khan, Daniya Ualiyeva, Asaf Khan, Nasib Zaman, Sanjeep Sapkota, Ayub Khan, Babar Ali, Dawood Ghafoor

**Affiliations:** ^1^State Key Laboratory of Respiratory Disease, Guangzhou Institutes of Biomedicine and Health, Chinese Academy of Sciences, Guangzhou 510530, China; ^2^University of Chinese Academy of Sciences, Beijing 100049, China; ^3^Center for Biotechnology and Microbiology, University of Swat, Mingora, Pakistan; ^4^Department of Herpetology, Chengdu Institute of Biology, Chinese Academy of Sciences, Chengdu 610041, China; ^5^Ministry of Education, Key Laboratory of Cell Activities and Stress Adaptations, School of Life Sciences, Lanzhou University, Lanzhou 730000, Gansu, China; ^6^Computer and Software Technology, University of Swat, Mingora, Pakistan; ^7^Department of Optometry, Isra University, Islamabad, Pakistan; ^8^CAS Key Laboratory of Special Pathogens, Wuhan Institute of Virology, Center for Biosafety Mega-Science, Chinese Academy of Sciences, Wuhan, Hubei, China

## Abstract

Air pollution (AP) is one of the leading causes of health risks because it causes widespread morbidity and mortality every year. Its impact on the environment includes acid rain and decreased visibility, but more importantly, it also has an impact on human health. The rise of COVID-19 demonstrates the cost of failing to manage AP. COVID-19 can be spread through the air, and atmospheric particulate matters (PMs) can create a good atmosphere for the long-distance spread of the virus. Moreover, these PMs can cause lung cell inflammation, thereby increasing sensitivity and the severity of symptoms in COVID-19 patients. In this study, we emphasized the potential role of PMs in the spread of COVID-19. The relationship among COVID-19, PMs, and angiotensin-converting enzyme 2 (ACE2) (receptor involved in virus entry into lung cells and inflammation) was also summarized.

## 1. Introduction

**Figure 1:**
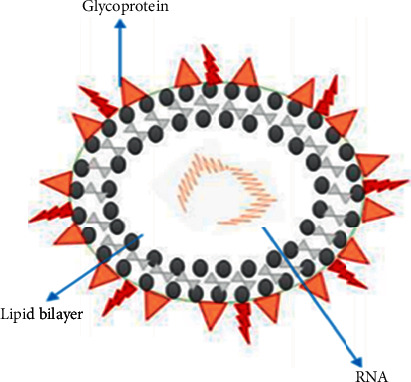
SARS-CoV-2 structure.

The emergence of SARS-CoV-2, which triggered an outbreak of coronavirus disease 2019 (COVID-19) in China, has posed significant public health risks worldwide [1, 2]. Coronaviruses are members of the Nidovirales order, which includes large, positive-sense enveloped RNA viruses classified into four genera: *α*, *β*, *γ*, and *δ* [3]. The novel SARS-CoV-2 is a crown-like, enveloped, positive-sense single-stranded RNA (+ssRNA) virus that belongs to the genus–coronavirus (Figure 1) [4, 5]. The coronaviruses use glycoprotein (S) homotrimeric spikes to enhance binding and entry into host cells. These spikes are core antigens present on the virus's surface and target neutralizing antibodies during the infection. Glycoprotein (S), produced as a single polypeptide of approximately 1300 amino acid precursors, is a class I virus fusion protein, so it can be used for viruses to enter cells. The binding of the virus to the bound receptor can enhance the cell surface density of the virus particle and support the interaction with the fusion receptor, for example, the binding of HCoV-OC43 and bovine coronavirus to N-acetyl-9-O-acetylneuraminic acid [6], the binding of HCoV-NL63 and SARS-CoV to heparan sulfate proteoglycan [7, 8], and the binding of HCoV-HKU1 to O-acetylated sialic acid [9]. In some cases, the attachment of virus particles to host cells is terminated [10]. In other cases, the decrease of adhesion receptors leads to a short interaction between the virus and the host cell, causing a significant decrease in virus infectivity [8, 11, 12]. Coronaviruses use diverse fusion receptors, except for HCoV-NL63, similar to HSARV-NL63, using human ACE2. In comparison, most alpha-coronaviruses use aminopeptidase N (CD13) to enter cells [13]. According to some studies, HCoV-OC43 uses HLA class I molecules or sialic acid [14]. MERS-CoV uses dipeptidyl peptidase 4 (DPP4 or CD26) [15], and the receptor for HCoV-HKU1 is still mysterious [9].

### 1.1. Emergence and Global Tally of SARS-CoV-2

In late December 2019, some native health centers in Wuhan, China, reported groups of patients with pneumonia of unfamiliar sources. Later, they found that these were epidemiologically related to wholesale seafood and wet animal [16]. Then, on 11 March 2020, the World Health Organization (WHO) publicized a pandemic of novel coronavirus (SARS-CoV-2) [17]. In late April 2021, more than 141,139,450 people contracted COVID-19 disease, and about 3,018,840 lost their lives. Still, the disease is upsurge, while scientists try to identify the mode transmission and possible correlation between the spread of viruses and air pollution. Thus, it is important to determine the possible role atmospheric particles play in the spread, morbidities, and mortalities of the coronavirus.

### 1.2. COVID-19 Possible Evidence of Airborne Transmission

**Figure 2:**
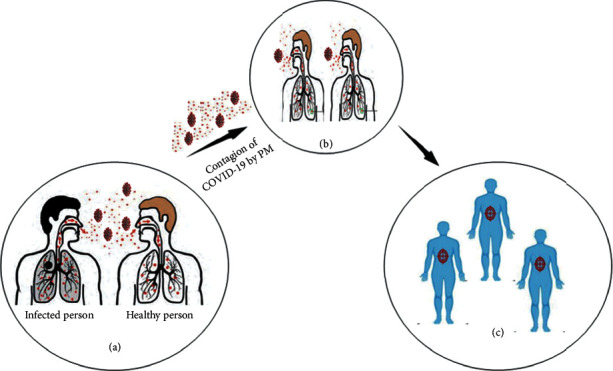
Illustration of COVID-19 contagion through PMs. (a) By direct sneezing (2 m). (b) Distantly infected individuals through PM (6 m or beyond). (c) Infected individuals.

Several scientific reports on the spread of viruses in humans have shown that the upsurge in infections is associated with the concentration of particulate matters (PMs) in the air (Figure 2) [18–20]. The well-known fractions of PMs are PM2.5 and PM10 [21,22], which act as carriers for a variety of chemical and biological contaminants (including viruses). Viruses can be adsorbed to PMs consisting of solid and liquid particles through coagulation, which can stay in the air from minutes to weeks. These particles or adsorbed biological contaminants may diffuse into the atmosphere and be transported over long distances. However, certain environmental parameters determine virus inactivation: the increased temperature and solar radiation can accelerate the inactivation level, while the relatively high moisture in the air can sustain the virus spread rate [23]. In support of PMs spreading the virus, new systematic reports have emphasized the association between the transmission of the virus in exposed populations and the level of PMs in the atmosphere. For instance, according to Chen et al., in 2010, due to Sahara dust, the extremely pathogenic avian influenza virus (H5N1) and environmental influenza may be transported over long distances [24]. The authors evidenced a high concentration of influenza A virus in the environment during the sandstorm in Asia compared to in the background day. Ye et al. reported that respiratory syncytial virus (RSV) infection in Chinese kids was related to high environmental temperature and airborne pollutants in 2016 [25]. Several other reports have proven that RSV causing pneumonia in children in most parts of the world is due to PMs transportation. The high infection rate of RSV was found to be positively correlated with PM2.5 (*r* = 0.446, *P* < 0.001) and PM10 (*r* = 0.397, *P* < 0.001). Additionally, Chen et al. in 2010 presumed that the prevalence rate of the virus is related to exposure to high concentrations of PM2.5 in the atmosphere of China [26]. More precisely, from October 2013 to December 2014, data on the number of daily measles patients and PM2.5 concentration were gathered from 21 Chinese cities, and the authors found that a 10 *μ*g/m^3^ increase in PM2.5 was significantly related to an escalation in the measles incidence rate. Finally, it is recommended to apply PM reduction strategies to slow the spread. Furthermore, the latest study conducted by Peng et al. in 2020 showed that people exposed to high PMs were considerably affected by measles in Lanzhou city of China [27]. Therefore, the authors suggested developing an effective PMs concentration reduction strategy to reduce the population's potential risk. According to the brief introduction of the above reports, it can be concluded that PM2.5 and PM10 can be active carriers for virus transportation, spread, and propagation of COVID-19.

### 1.3. Objectives of the Study

This article emphasizes two assumptions. Firstly, similar to other viruses, COVID-19 can also be spread through the air, and PMs can be used as a carrier through aerosols to extend the virus and increase its spread. Secondly, PMs may cause damage to lung cells and increase inflammation. This increase in inflammation may cause a rise in mortality rate and disease severity in the most contaminated regions. The virus attaches to the ACE2 receptor to enter cells; ACE2 produces anti-inflammatory peptides, whose overexpression in inflammation is caused by exposure to PMs exerting its effects, thus accelerating the possibility of virus entry into cells. These assumptions are communally exclusive and are significant starting points for upcoming analysis, aiming to explain the positive association between air contamination and the transmission of COVID-19.

## 2. Methods

We searched for articles on topics of interest in Google Scholar, PubMed, Sci-Hub, WHO, and PubChem to analyze scientific literature. Different terms were used to facilitate the search for related articles from search engines and databases. Keywords such as COVID-19, SARS-CoV-2, particulate matters, air pollution, the impact of air pollution on humans and the environment, the participation of particulate matter in COVID-19 transmission, and several other terms were used. The authors searched the reference list of the included studies to ensure that the literature was covered. Finally, we analyze all full-text reports to see if they meet the inclusion criteria. The majority of included articles were emphasizing the harmful effects of COVID-19 on humans. Most of the articles included in the study have been published recently, although some earlier published articles are also cited to clarify the nature of the particles, problems, and their impact on humans because no applicable latest scientific works have been published on related topics.

## 3. Results

### 3.1. Potential Role of PMs in the Spread of COVID-19

The impact of PMs fumes and the transmission of the virus among people have been researched and analyzed in different world regions with numerous incidents. The infectious disease of COVID-19 transmission is related to a higher degree of air pollution. For example, in three regions of the world, several individuals are infected with COVID-19: China, the epicenter of the pandemic; Italy; and the United States, where high levels of air pollution are a correlation among these nations. This is why recent research has emphasized the possible association between air pollution and COVID-19 infectivity.

Two levels should be evaluated for the assessment of this potential association: (i) elevated rate of air contamination in the past making people vulnerable to COVID-19 (long-term exposure) and (ii) sensitivity to viruses related to high levels of air contamination during the contact (short-term exposure). For instance, it has been known that regular PMs contact with the atmosphere will escalate hospitalization and mortality, mainly distressing the cardiac and respirational systems and causing various diseases, such as cancer [28]. Moreover, it is estimated that these pollutants cause 2 million premature deaths from acute respiratory diseases every year in the world [29, 30]. To assess the long-standing fair assumptions, Pansini and Fornacca studied the geographic level of infection that has expanded, and then they linked the data with the annual air quality index using annual averages. Observations were made from the Sentinel-5 satellites revolving around China, Italy, and United States. Numerous contaminants (PM10, PM2.5, sulphur dioxide, carbon monoxide, nitrogen dioxide, and ozone) were analyzed. A significant positive association was found in each country among COVID-19 infections and air quality variables and poor air quality, that is, high PM2.5, carbon monoxide, and nitrogen dioxide, which was also correlated with higher mortality rates [31]. Concerning Italy, Fattorini and Regoli, and Conticini et al. have found that circulation of air pollutants (PM2.5, PM10, nitrogen dioxide, and ozone) in northern Italy has surpassed regulatory limits over the last four years. Thus, people infected with the infectious disease COVID-19 have suffered from high levels of air pollution for a long time. These conclusive data show that, in 71 Italian provinces, air quality is significantly related to COVID-19 cases, further demonstrating that long-term interaction with air pollution may be a favorable environment for airborne transmission of the virus [32, 33].

**Figure 3:**
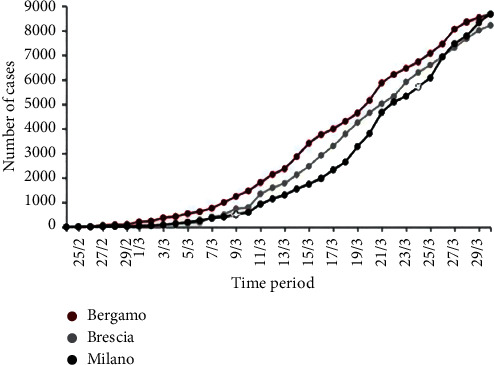
Illustration of the growth in the number of COVID-19 positive cases in Bergamo, Brescia, and Milan from late February to late March 2020 (generated and modified from [38]).

To conclude, in China, France, Germany, Iran, Italy, Spain, the United Kingdom, and the United States, Pansini and Fornacca have associated annual satellite and ground air indexes. They found a statistically relevant positive correlation between high levels of air pollution and infections with COVID-19. The correlation between the worst air quality and the presence of COVID-19 and its caused fatalities was the most apparent in Italy [34]. Wu et al. currently applied a zero-inflated negative binomial mixed model (for confounding factors), evaluating the relationship between long-term PM2.5 exposure and COVID-19 fatality in the United States, and found statistically significant results. According to their study, the COVID-19 mortality rate rose by 15% for every 1 ug/m3 and rose in long-term exposure to PM2.5. This article's consequences demonstrated that continuous exposure to air pollution is more likely to cause more COVID-19 severe results. The findings were consistent with the well-known relationship between exposure to PM2.5 and several cardiac and respiratory comorbidities, which substantially increased the risk of fatality in COVID-19 patients [35]. The site paper proposed by the Italian Association of Environmental Medicine (SIMA) claims that PMs are the essential carrier for promoting the spread of COVID-19 concerning the effect of temporary exposure to PMs and virus transmission in the population [36]. Moreover, increased and rapid transmission of COVID-19 in the Po Valley (the most contaminated location in Europe) could be related to prepandemic PMs concentrations in Lombardy [37]. For instance, Bergamo was one of the most infected Italian cities (Figure 3). Concentration of PMs was more significant between January and February 2020 than the allowed annual mean concentration of PM10 and PM2.5. The limit for PM10 was 40 *μ*g/m3, and the limit for PM2.5 was 25 *μ*g/m3, while the central unit of the city (via Meucci station) detected during those two months that the average daily PM10 concentration was 44.28 *μ*g/m3 and the daily average of PM2.5 concentration was 38.31 *μ*g/m3, both higher than the maximum limit set. The number of days when the normal PM10 and PM2.5 concentration values surpass the acceptable threshold was 33 and 44, respectively [39].

There have been numerous studies supporting this evidence. In Italy and China, Frontera et al. analyzed air quality during the most virulent time of COVID-19; the PM2.5 and nitrogen dioxide levels were exceptionally high [39]. Marteletti and Marteletti reached the same conclusion [40]. Based on a recent SIMA investigation, these authors hypothesized that the atmosphere is rich in air pollutants, coupled with particular climatic conditions, which may, as carriers, encourage longer persistence of virus particles in the air, thereby facilitating indirect transmission.

### 3.2. PMs Can Damage Pulmonary Cells and Cause Inflammation and Oxidative Stress

Many epidemiological reports stated various causes for the pollution levels and hospitalization, including respiratory diseases. At the same time, there has also been an increase in mortality from numerous viral diseases. To evaluate PMs' role in spreading viruses, it is also vital to diagnose how exposure to contaminants increases the susceptibility and severity of these diseases. PMs, as described earlier, have a tiny size so that it can be sucked in. Although frequent inhalation of these particles can harm lung conditions, constant contact with PMs can also cause systemic damage [18]. The pollution acquaintance was also associated with the SARS-CoV-1 virus's high death rate. Via the air pollution index (API), Cui et al. have demonstrated that the probability of death is twice as high in areas with high air pollution indexes as in areas with low air pollution indexes. Similarly, the possibility of an increased risk of demises from SARS was 84% in areas with a medium API. The authors concluded that prolonged exposure to PMs increases mortality also with viruses [41]. After proving that there is a positive correlation between exposure to PMs and respiratory virus infections, the mechanism of exposure to these substances that may affect the susceptibility of the subject to infection and the immune response must be analyzed because respiratory cells are the prime target for PMs and also a primary target for respiratory viruses. If the subject is exposed to PMs for a long time, pathogens will invade the compromised cells. It has been shown that, in humans and experimental models, exposure to PMs can induce two mechanisms in the lungs:

1. Oxidative stress: contact with these pollutants can induce free radicals, leading to cell damage
2. Inflammation: PMs cause stimulation of an immune response so that cells reach the condition of inflammation, which stimulates several pathways that are functional at the inflammation level [42]

The potential of pollutants to affect resistance by restraining the antiviral response of exposed subjects is another mechanism to note. The vital role of an inflammatory reaction is that macrophages can ingest and destroy foreign substances, including microorganisms, in the cytoplasm [43]. However, some reports have determined that exposure to pollutants can decrease phagocytic exposure and the tendency of macrophages to avoid proper inactivation of the virus [44].

In short, exposure to contaminants changes the pulmonary cells' immune response and leads to increased oxidative stress and inflammatory stress. This cellular disorder promotes viral attacks and raises the frequency of viral infections in exposed subjects. For instance, owing to the onset of high PM10 contamination, viral pneumonia often increases. A study in 1999 has assessed that how PM10 alters the inflammatory response of the RSV. The immunity to the virus and PM10 detected at the same time at elevated levels is less successful than a single immune response to RSV protection, thereby reducing the response to the virus [45]. The PO area is one of the most polluted regions of the country in northern Italy, with many factories and a particular region between the Alps and the Apennines. Samples of PM10 and PM2.5 were obtained at Torre Sarca, Milan, and administered to mice during 2010 to examine the damage caused by exposure to these particles in the lungs. The microbiological study indicates that pathogens are adsorbed to particles [46], and PMs cause an inflammatory reaction in alveolar cells and lungs. The elevated levels of proinflammatory cytokines such as tumor necrosis factor- (TNF-) alpha and IL-6 have been identified [47]. It should also be noted that IL-6 triggers the most severe inflammatory storm in COVID-19 patients. Studies have shown that systemic disease patients are at elevated risk of severe types of infection with COVID-19 [48]. It can also not be ruled out that the virus would promote the cells that are already defective. Exposure to toxins harms the lungs and heart, and defects in these cells can lead to a more complicated prognosis.

### 3.3. COVID-19 and Inflammation

The coronavirus may cause mild or very severe pathological reactions. It can cause significant respiratory damage and may even be fatal if these viruses invade the deep part of the respiratory tract. At the same time, inflammation is an integral part of our immune system. For SARS-CoV-1, however, an overreaction and disorder with a “cytokine storm” have been observed in patients with the severe disease [49]. Various pathological symptoms can also be caused by the novel COVID-19 virus, from mild colds and fevers to severe illnesses such as pneumonia. It worsens as it causes acute respiratory distress syndrome (ARDS), causing respiratory failure and fluid, which accumulates in the lungs, and significantly decreasing the blood oxygen level. 10% of COVID-19 cases can enter this pathological disorder, requiring mechanical ventilation, and it is even fatal in some cases. However, for the aged individuals and patients with other contemporary pathologies, the course of the disease is more complicated [50]. As an indication of inflammation, a large amount of IL-6 was found in patients' blood with extreme symptoms of COVID-19. An inflammatory storm has been observed; the immune response of infected cells releases inflammatory signaling cytokines, but excessive proinflammatory signals may damage pulmonary epithelial cells [51]. The first clinical trial of the medication used to treat COVID-19 has been approved by the Italian Medicines Agency (AIFA).

One way was to reduce inflammation caused by an immune response that was excessively high. In reality, several individuals focused on anti-inflammatory drugs targeting anti-inflammatory storms in these trials. Tocilizumab, for instance, is a monoclonal antibody that, by inactivating it, can bind to the IL-6 receptor. Hindering the IL-6 transduction pathway can also decrease the inflammatory state [52]. Inflammatory storms have some vulnerability due to the existence of other pathologies. Specific pathogenesis patients start with higher inflammatory cytokines, while the body imbalance is triggered by advanced age and appears proinflammatory. Another possibility is that a specific genetic predisposition may occur. We can also presume that over time, after COVID-19 infection, subjects exposed to the most PMs are prone to this cytokine storm, so the pathological process is more complicated. This assumption may demonstrate the positive relationship in some polluted areas among COVID-19, PMs concentration, and high mortality (such as Lombardy, Italy).

The study by Conticini et al. in March 2020 reveals a significant relationship between air pollution and COVID-19 lethality in different regions of Italy, with an incidence of 12% in Lombardy and Emilia-Romagna on 21 March 2020 and 4.5% in the rest of Italy. Contaminant exposure makes individuals more vulnerable to severe respiratory diseases in these regions, making it convenient to include a cofactor [33].

## 4. Discussion

SARS-CoV-2 has a spike protein that binds it to the receptor ACE2 present on the cell. Via endocytosis, the virus enters, and cells are infected. ACE2 is a membrane enzyme in the lungs, arteries, heart, kidneys, and intestines. It catalyzes peptides with vasoconstriction and angiotensin 2 into angiotensin 1–7 [53]. It is speculated that blood pressure rises because of age. Furthermore, our body expresses more ACE2 on the cell membrane as a compensatory response. This increase in expression, however, also improves the targets that enable the COVID-19 virus to enter. Certain medications increase ACE2 expression for hypertension, so this association should be confirmed. Since there are also anti-inflammatory properties of angiotensin 1–7 peptide, ACE2 also has another task. Activation of ACE2 has been shown to reduce hyperoxia-induced lung injury and suppress inflammation and oxidative stress. ACE2 can suppress the activated B cell nuclear factor kappa light chain enhancer (NFKB) (inflammatory response pathway) intracellular signal and activate the Nrf2 associated with the erythroid nuclear factor 2 signal. Activation of anti-inflammatory response is a defense mechanism against ROS [54]. The rise in ACE2 would therefore increase the probability of a COVID-19 attack. However, on the other side, by binding to ACE2, which seems to be very necessary for immune protection and defense against inflammation, the virus prevents its action. This is the main reason for the COVID-19 deaths. Oxidative stress and inflammation are caused by repeated exposure to PM2.5, and both activation mechanisms are as mentioned above: Nrf2 reacts to oxidative stress and NFKB, which causes inflammation [55]. In 2018, Lin and coauthors reported that exposure to PM2.5 causes acute lung injury (ALI) in mice, resulting in increased inflammation and higher levels of cytokines. In both wild-type (WT) and ACE2 (knockout) mice, the effect of PM2.5 was shown. It was noted that lung damage improved after a few days in wild-type mice; the improvement in mice lacking ACE2 was not significant. This confirms ACE2's fundamental role in shielding our cells from PM2.5's proinflammatory effects. However, importantly, in wild-type mice, exposure to PM2.5 causes a significant increase in ACE2 [56].

**Figure 4:**
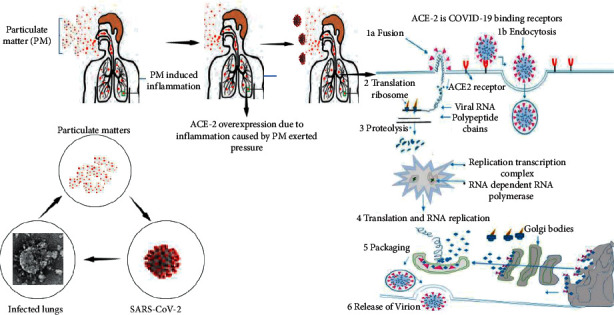
Illustration of PMs, ACE2, and inflammation relationship.

In conclusion, ACE2 is very significant because (a) the Nrf2 (anti-inflammatory) pathway is activated and the NFKB (inflammatory) pathway is switched off to prevent inflammatory response disorders; (b) COVID-19 modifies this mechanism by combining it with ACE2; (c) ACE2 is overexpressed after exposure to PM2.5, thereby increasing the risk of COVID-19 infection, where ACE2 is the key to the virus's entry (Figure 4).

## 5. Conclusion

In short, the available information about the global spread of SARS-CoV-2 supports the hypothesis of the droplet model in the air with long distances between people. The potential coalescence phenomenon that occurs between the nucleus of the droplet and the particulate matter is that it is considered reasonable, especially under favorable environmental conditions, to stabilize the droplet core. Therefore, based on the evidence discussed above, it is reasonable to describe this virus transmission model as a “super transmission event,” as evidenced by the high estimates in northern Italy at the beginning of the pandemic. Therefore, it is mandatory to adopt a face mask policy during the pandemic, when gradually returning to everyday life is to be expected. In the regular use of masks, the distance between people can be reduced to 2 m. The most common mask that covers the upper respiratory tract of humans does not allow the ACE2 protein placed in the mucous membrane of the mouth and nose to come into contact with the virus. Under outdoor conditions, droplet nuclei are more dispersed in the atmosphere (even if they aggregate as particles), and without a mask, even if the distance between people is shorter than 10 meters, it can ensure low infection risk. Finally, scientific evidence on the correlation between PM levels and the spread of SARS-CoV points to opportunities to strengthen strategies to reduce anthropogenic PM emissions and reduce citizens' exposure to PM and uncontrolled aerosols.

## Abbreviations

COVID-19: Coronavirus disease 2019
PMs: Particulate matters
ACE2: Angiotensin-converting enzyme 2
SARS: Severe acute respiratory syndrome
CoV: Coronavirus
WHO: World Health Organization
SARS-CoV-2: Severe acute respiratory syndrome-coronavirus-2
+ssRNA: Positive-sense single-stranded RNA
HCoV: Human coronavirus
HLA: Human leukocyte antigen
MERS-CoV: Middle East respiratory syndrome-coronavirus
DPP4: Dipeptidyl peptidase 4
RSV: Respiratory syncytial virus
API: Air pollution index
TNF: Tumor necrosis factor
IL-6: Interleukin-6.
